# Coughing in Small Animal Patients

**DOI:** 10.3389/fvets.2019.00513

**Published:** 2020-01-21

**Authors:** Brisa M. Hsieh, Alicia K. Beets

**Affiliations:** ^1^Internal Medicine, Southern Arizona Veterinary Specialty and Emergency Center, Tucson, AZ, United States; ^2^Internal Medicine, MedVet Medical and Cancer Centers for Pets, Metairie, LA, United States

**Keywords:** cough, respiratory, cardiac, veterinary, canine, feline

## Abstract

Cough is one of the most common complaints in human medicine. In veterinary medicine cough is equally frequent and plays a significant role in an owner's perception of their pet's quality of life. In human and veterinary medicine, therapy for chronic cough is often ineffective. The complexity of the cough pathway and species differences have made it difficult to develop an effective antitussive agent for veterinary patients. The few effective antitussive agents currently available are associated with significant adverse effects. Fortunately, several promising drugs currently being studied in human clinical trials may offer options for use of novel antitussive therapies in small animal patients. This article reviews current understanding about cough pathophysiology, diagnostic strategies used to uncover underlying etiology of cough, and examines available options for controlling cough, including novel antitussive therapies used in human medicine.

## Introduction

Cough is a vital reflex that serves as a defense mechanism for clearing the airways of foreign material, enhancing the mucociliary escalator, and protecting airways against inadvertent aspiration of material from the oral cavity (1). In veterinary practice, cough is an important clinical sign that indicates an underlying disease process. Despite its obvious protective function, chronic, non-productive cough may lead to airway mucosal damage and other respiratory compromise as well as patient discomfort and reduced quality of life (2). There are several ways to categorize a cough but perhaps the most clinically useful ways include determining the length of time (acute or chronic) and whether the cough is due to respiratory or cardiac disease. If cough is related to respiratory disease, it is necessary to determine the anatomic localization within the affected respiratory tract (e.g., upper or lower respiratory tract, pleural cavity, or pulmonary parenchyma) (3). Depending on the etiology and type of cough, treatment may or may not be indicated. Treatment of the underlying problem rather than cough suppression is the most effective strategy for addressing acute cough. When an underlying cause is not identified or cannot be eliminated or the cough duration is longer than 2 months, antitussives are indicated to manage cough and restore acceptable quality of life for the patient and owner (2). For patients with chronic cough that persists despite appropriate management of underlying disease antitussives are essential to maintain an adequate quality of life for the patient and owner (4). Unfortunately, effective antitussive therapy is a need largely unmet in human and veterinary medicine (5, 6). This article reviews current understanding about cough pathophysiology, diagnostic strategies used to uncover underlying etiology of cough, and examines available options for controlling cough, including novel antitussive therapies used in human medicine.

## Types Of Cough

The European Respiratory Society (ERS) defines cough “as a forced expulsive maneuver or maneuvers against a closed glottis that are associated with a characteristic sound or sounds.” Cough is an important protective reflex to enhance airway clearance and prevent aspiration (5). The ERS defines chronic cough as lasting at least 8 weeks in adults and 4 weeks in children (7). A cough is considered chronic in veterinary patients if it has been present for at least 2 months (8). In the clinical setting, characterization of cough can assist in determining its etiology. Cough can be categorized as acute, if present for <2 months, or chronic based on the duration of signs. Descriptors (e.g., dry, moist, honking, productive) may be helpful for localizing airway pathology or suggesting cough etiology (Table 1). For example, a moist cough that produces sputum indicates the presence of excess fluid in the airways and suggests pneumonia or edema as possible etiologies. Commonly, owners do not recognize a productive cough because the dog or cat swallows expectorated material (8, 9). A cough can also be classified as purposeful, warning, or nuisance (Table 2) and can have cardiac or respiratory causes. Taking a thorough history, including a description of the cough (soft, honking, or gagging), presence of any respiratory difficulty or abnormal breathing sounds (wheeze, stridor, or stertor), and circumstances that elicit the cough (excitement, eating, or drinking) is vital to classify a cough (3, 10). In some animals, especially cats, a thorough patient history and careful questioning of the owner are needed to distinguish cough from vomiting or retching (10).

**Table 1:** Classification of cough: productive vs. non-productive.

**Type of cough**	**Characteristic of cough**
Productive	Moist Low-pitched Material expectorated from trachea into pharynx—usually swallowed in the dog or cat
Non-productive	Dry Harsh, high-pitched, honking Expectoration of mucus—possible but not characteristic

*Adapted from Rozanski and Rush (8) and King (9)*.

**Table 2:** Classification of cough: purposeful vs. warning vs. nuisance.

**Type of cough**	**Definition**	**Example**	**Treatment**
Purposeful	Beneficial cough Expulsion of material and air in airways Assists to eliminate underlying problem	Bacterial pneumonia—expel purulent material Inhaled foreign body—expel foreign material	Treat the underlying problem
Warning	Manifestation of a serious underlying disease process	Pulmonary edema Pulmonary neoplasia Pulmonary thromboembolism Lung lobe torsion Severe allergic respiratory disease	Immediate treatment needed of underlying condition
Nuisance	Cough reflex unnecessarily triggered	Airway collapse/obstruction Chronic sterile bronchitis Cardiomegaly causing tracheal/bronchial pressure Uncomplicated infectious tracheobronchitis Mild allergic airway disease	Cough suppression is indicated in addition to other treatments

*Adapted from Hoskins (10)*.

Coughing is a voluntary or an involuntary action (1, 11). Involuntary cough is stimulated by changes in airway pH, specifically caused by aspiration of gastric contents, and is transmitted through afferent A-δ fibers. Involuntary cough also occurs due to activation of unmyelinated vagal sensory C-fibers (12). Cough in cats and dogs is thought to be an involuntary reflex, although the immediate trigger is often not identified; voluntary cough is not recognized in small animals (8).

## Pathophysiology

Muscles of the diaphragm, thoracic cage, larynx, and abdominal wall work in concert to produce the forceful mechanical actions associated with the cough reflex. The cough response can be divided into inspiratory, compressive, and expulsive phases. Coordinated neural and muscle activation and intake of a large volume of air comprise the inspiratory phase. The compressive phase is characterized by a sharp increase in intrathoracic pressure against a closed glottis and is followed by the expulsive phase that involves rapid opening of the glottis and forceful ejection of air (13).

Afferent, central, and efferent pathways comprise the reflex arc that generates cough. The afferent pathway encompasses several branches of the vagal nerve (diaphragmatic, cardiac, and esophageal branches) and vagal sensory nerve fibers localized within the ciliated epithelium of the upper airway that have diffuse projections to the medulla. The central pathway coordinates afferent vagal signals with the cough center located in the pons and upper brain stem. The efferent pathway takes impulses from the cough center to the effector organs, namely the inspiratory and expiratory muscles and larynx (14).

Anatomical differences in afferent fiber distribution influence the response elicited by various cough stimuli. Proximal airways are most sensitive to mechanical stimulation, whereas the distal airways are more sensitive to chemical stimulants. Therefore, the expected cough response can often be anticipated based on the type of stimulation and the anatomical site affected (4).

**Figure 1:**
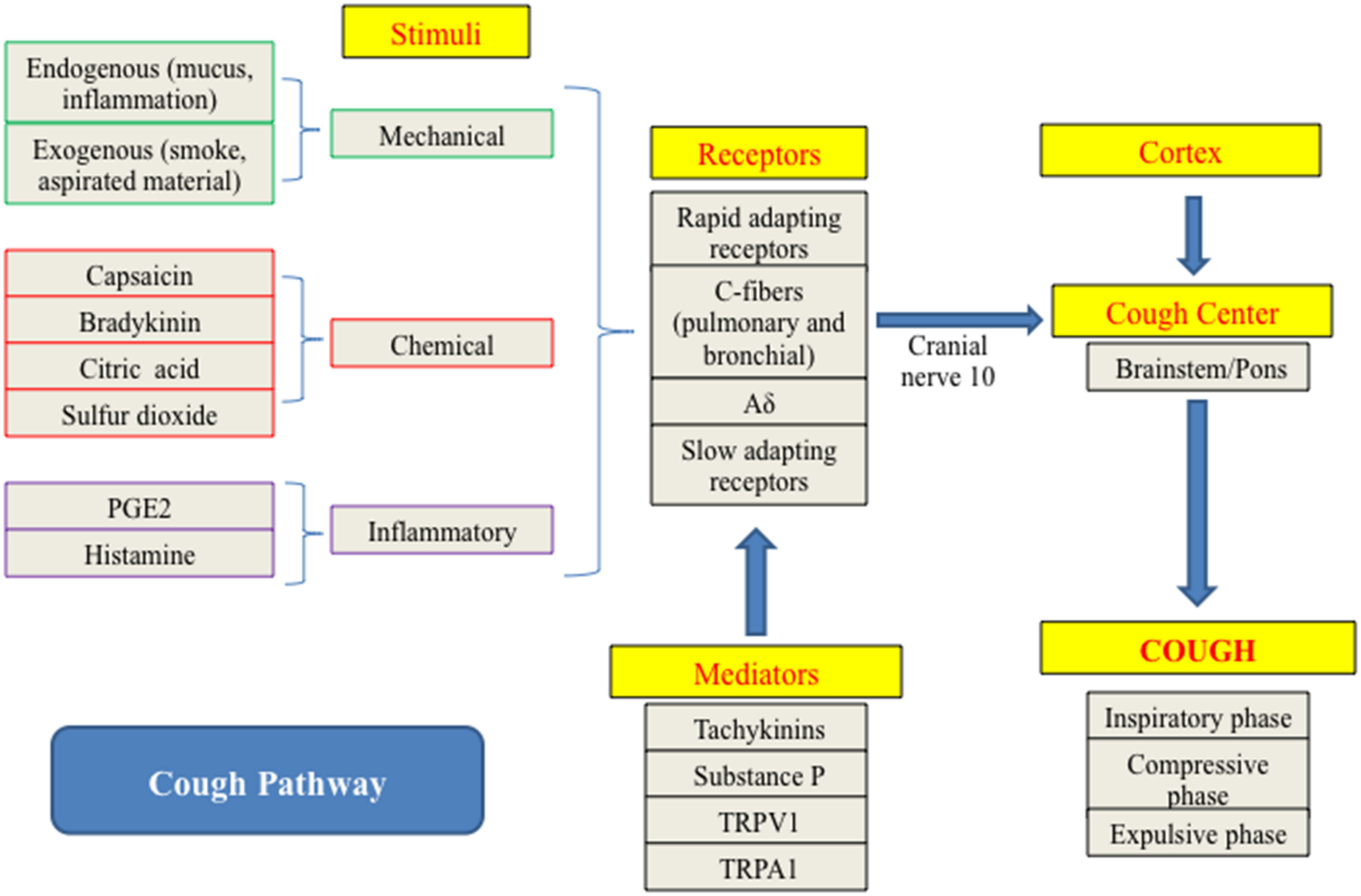
Pathway showing initiation and propagation of the cough response (8, 13, 15, 16).

As previously mentioned, cough can be initiated through mechanical, chemical, or inflammatory stimulation (15) (Figure 1). Endogenous stimuli, such as airway secretions and inflammation, and exogenous agents, such as smoke, aspirated material, or other inhaled foreign substances, both play a role in triggering the cough reflex (1). Specific disease processes can also amplify the response an individual has to particular stimuli. For instance, infection with *Bordetella bronchispetica* causes a marked increase in response of the rapidly adapting stretch receptors (RARs) lowering the cough reflex threshold (17).

The cough reflex is activated via peripheral or central stimulation. Peripherally, activation of C-fibers and cough receptors are the most common triggers for cough. Centrally, only two excitatory transmitters mediate cough, glutamate and neurokinins (NKs), most importantly neurokinin A (NKA). Glutamate is thought to be the primary excitatory neurotransmitter while NKs predominantly maintain a modulatory role. NK expression peripherally is restricted to capsaicin-sensitive nociceptors (16).

Various stimuli activate cough receptors including RARs, slow adapting stretch receptors (SARs), and C-fibers. RARs, which are myelinated, exist primarily within the mucosa of the tracheobronchial tree and respond to weak mechanical stimulation. When RARs are activated, bronchospasms and mucus production are stimulated via parasympathetic pathways. SARs, which are also myelinated, are most sensitive to mechanical forces, largely moderate lung inflation, and are mainly found within intrapulmonary airways. C-fibers constitute the majority of afferents within the airways and are unmyelinated. They are located in close apposition to the blood vessels and are divided into two types, pulmonary and bronchial. Unlike RARs and SARs, these receptors are insensitive to mechanical stimuli and less responsive to lung inflation (4, 11, 14). C-fibers are directly stimulated by bradykinin and capsaicin. Pulmonary C-fibers are located within the small peripheral airways and supplied by the pulmonary circulation whereas bronchial C-fibers are found within the larger airways, supplied by the bronchial circulation, and are most sensitive to chemical stimulants (11, 15). Additionally, C-fibers are important for bronchoconstriction and neural control of respiration (11, 18). Results of C-fiber activation include increased airway parasympathetic nerve activity, as well as bradycardia, hypotension, and apnea secondary to activation of the chemoreflex (14). All stimuli are transmitted via the vagus nerve to the cough center, where a cough is initiated. Direct input to the cough center from the cerebral cortex can also elicit a cough (11).

Transient receptor potential channels are a family of cation channels found on vagus nerve endings located in and below airway epithelium and are triggered by pH, osmolarity, temperature, inflammatory and mechanical stimuli, and environmental irritants (15, 19, 20). The two most significant channels to evoke a cough are transient receptor potential vanilloid receptor subtype 1 (TRPV1) and transient receptor potential ankyrin 1 (TRPA1) channels. TRPV1 is stimulated by pollutants, capsaicin, allergens, bradykinin, and acids. TRPA1 is stimulated by smoke, ozone pollutants, and bradykinin. Once initiated, these stimuli activate the C-fibers. If a sufficient depolarization is created from the initial event, voltage-gated sodium channels open and an action potential travels to stimulate release of glutamate, substance P (SP), and NKs from neurons. Glutamate acts on N-methyl-D-aspartate (NMDA) and non-NMDA receptors while SP acts on NK_1_ receptors and NKA acts on NK_1_, NK_2_, and NK_3_ receptors (15, 16). The nucleus tractus solitarius, which also receives direct input from other afferent nerves, is then stimulated to produce a cough (16).

Some studies demonstrate that dogs may have different pathophysiology of the cough reflex than people, cats, and guinea pigs. Guinea pig models are commonly used for airway research as guinea pigs respond to a variety of antitussives and cough similarly to humans (19). Boyle et al. demonstrated methods successful in inducing cough in cats, guinea pigs, and humans are not successful in dogs (6). A connection between cigarette smoking, environmental tobacco smoke, and wood smoke have all been associated with cough and pulmonary disease in people but the same association has not been established in dogs with chronic cough (21). Other studies have associated environmental tobacco smoke as an increased risk for lung and nasal cancer in dogs (22, 23). One study demonstrated bronchoalveolar lavage fluid obtained from dogs exposed to environmental tobacco smoke had anthracosis with increased macrophages and lymphocytes (24). Several laboratory studies have documented airway epithelial changes and airway inflammation in dogs with exposure to direct cigarette smoke (25–27). Based on these varying results, there is a gap in the knowledge regarding the effects of airway irritants and pulmonary disease in dogs and a lack of complete understanding of cough pathophysiology in veterinary patients.

## Respiratory vs. Cardiac Cough

Cough is a feature of disorders originating from the upper or lower respiratory tract. Diseases from the upper respiratory tract associated with cough include among others, rhinitis, laryngeal disease, tracheal collapse, and infectious tracheobronchitis. Common lower respiratory tract diseases include chronic bronchitis, bronchomalacia, pneumonia, idiopathic pulmonary fibrosis, eosinophilic bronchopneumopathy, lungworm disease, and others. Some etiologies, such as inhaled foreign material and neoplasia, can affect both the upper and lower airway tracts. Disease in the pleural space, such as lymphadenopathy or mediastinal mass can produce cough through airway compression or other mechanisms that indirectly affect the airways (3).

Coughing is frequently reported in dogs with cardiac disease. However, it is not a common clinical sign in cats with cardiac disease. Enlargement of the left atrium can compress the mainstem bronchi and stimulate cough that is commonly observed in dogs with mitral valve disease. In dogs with congestive heart failure, edema fills the airways and pulmonary venous distention stimulates juxtapulmonary receptors. When the receptors are stimulated, reflex bronchoconstriction and increased mucus secretion occur, resulting in a cough. Coughing that occurs secondary to heart disease but in the absence of congestive heart failure can be challenging to differentiate from a cough due to large airway disease. It is also possible that pulmonary and cardiac causes contribute to cough in some disorders (28).

## Cough Localization

On presentation, a general exam can help localize the cough. After observing the patient for signs of respiratory abnormalities and cough, vitals should be obtained. Anecdotally, if the temperature is high normal or elevated, one may be more likely to think primary respiratory disease as an underlying cause, where as if the body temperature is low, especially in cats, one may lean more toward cardiac disease causing decreased perfusion leading to hypothermia as an underlying cause of the cough (28).

After general observation and vitals are performed, it is important to identify the presence of any other abnormalities. Look for signs of nasal discharge and sneezing and evaluate nostril airflow and the presence of facial asymmetry. Listen for any abnormal respiratory sounds including stridor, stertor, or wheezes. Auscultation over the larynx and trachea should be performed, especially if the patient has abnormal airway sounds or difficulty breathing (3). Laryngeal disease may be present if the patient has inspiratory stridor (29). A patient with nasal discharge, abnormal nostril airflow, and/or sneezing is more likely suffering from nasopharyngeal disease (30). If a patient is cachexic, cardiac disease or neoplasia may be higher on the differential list while an obese patient may be more likely suffering from respiratory disease (3).

Auscultation of the heart and lungs is essential to evaluate the patient for abnormal pulmonary sounds, heart murmurs, or arrhythmias. A patient without a heart murmur will less likely have cardiac disease and be more likely to have a primary respiratory problem. Pulmonary crackles can be present due to cardiogenic as well as non-cardiogenic pulmonary edema, pulmonary fibrosis, and inflammatory pulmonary diseases (28). A tachycardic patient with an arrhythmia may be more likely to be in congestive heart failure while a patient with bradycardia or a respiratory sinus arrhythmia is more likely suffering from respiratory disease (3, 28). If the patient has jugular pulses, hepatomegaly, and/or ascites, right-sided heart failure needs to be considered, as cardiac disease is a probable cause of cough (28). Pleural effusion can cause decreased heart and lung sounds while only reduced cardiac sounds may be due to pericardial effusion (28). Commonly, small breed dogs have both chronic mitral regurgitation and airway disease. A concurrent systolic heart murmur can be heard in at least one third of dogs with tracheal collapse (29). If tracheal disease (e.g., tracheal collapse or *Bordetella bronchiseptica*) is the primary problem, tracheal sensitivity may be present and a cough can often be induced on physical exam by palpation of the trachea, which is best left to do at the end of the exam (3, 29). However, palpation-induced cough is not a definitive indicator for primary tracheal disease as dogs with other respiratory diseases and even normal dogs may cough during tracheal palpation (10). A loud cough is generally due to large airway disease and a soft cough is often indicative of lower airway disease (3). Localizing cough is the first step to help discover cough etiology and direct diagnostic steps, which will aid in selecting best treatment options.

## Diagnostics

Thoracic imaging, pro-B-type natriuretic peptide (proBNP), laryngeal exam, bronchoscopy, airway cytology and culture are effective tests to help identify the underlying cause for a patient's cough. Baseline lab work including a complete blood count, serum biochemical profile, urinalysis, and heartworm disease testing is recommended to evaluate the patient's general health, especially if the patient may be undergoing general anesthesia for more invasive diagnostics. When recommending diagnostics for a patient with cough, least invasive testing should be completed first (31, 32). It is imperative to stabilize a patient with respiratory distress prior to performing diagnostic testing.

## Thoracic Imaging

Thoracic radiography, fluoroscopy, thoracic ultrasound, or thoracic computed tomography (CT) may be useful to identify the cause of cough. Thoracic radiography is generally performed first and allows evaluation of respiratory structures (33). The trachea is evaluated for signs of collapse, compression, or obstruction. The vertebral heart score can be calculated and the pulmonary vessels and lung parenchyma evaluated for evidence of congestive heart failure. The pulmonary parenchyma, bronchial tree, and pleural space can be evaluated for changes consistent with pulmonary infiltrates or masses, bronchitis or bronchiectasis, or effusion (29, 32). Three radiographic views of the thorax (ventrodorsal, right lateral, and left lateral) are essential to fully evaluate the pulmonary parenchyma and help rule out solitary lesions that may be contributing to the cough (34). Inspiratory and expiratory cervical radiographs may be beneficial if laryngeal or tracheal disease is high on the differential list (31).

Fluoroscopy is needed for the diagnosis of cough caused by dynamic airway abnormalities. Tracheal collapse is the most common etiology responsible for dynamic cough in dogs. Tracheal collapse can be suspected based on radiography in 59–84% of cases (29). Because the cervical trachea collapses during inspiration and intrathoracic trachea collapse occurs during expiration, radiographic diagnosis is improved by evaluation of multiple lateral views obtained during inspiratory and expiratory phases of respiration. However, radiography is generally an insensitive diagnostic method since collapse at the level of the carina is often not detected and tracheal collapse can appear to be less severe (31). Fluoroscopy is indicated when tracheal collapse is suspected but is not confirmed using plain radiography or for anatomic localization of collapse prior to intervention (29, 31). In one study, 8% of tracheal collapse cases diagnosed by fluoroscopy were not detected by radiography. In the same study, the site of collapse visualized radiographically was incorrect in 44% cases that were examined using fluoroscopy (31, 35). Fluoroscopy can also be used to detect lower airway collapse (31).

Thoracic ultrasound is indicated to further evaluate abnormal radiographic findings including cranial mediastinal masses, pleural effusion, pulmonary infiltrates or solitary pulmonary masses. Radiographs typically guide the location a thoracic ultrasound is performed. Ultrasound-guided fine needle aspiration (FNA) or needle biopsy is used to obtain samples for cytology, histology, or culture. Mediastinal masses or diaphragmatic hernias may be masked by pleural effusion on radiographs and can be visualized with ultrasound. Echogenic characteristics of fluid can suggest the presence of fibrin, protein, and/or cells. If pleural effusion is present, the diaphragm can be assessed for abnormalities. While thoracic ultrasound can be helpful when combined with radiographs, there are limitations. The large acoustic impedance mismatch between the ultrasound beam and air and bone limits the diagnostic use of ultrasonography to evaluation of structures near the thoracic wall, utilizing an intercostal, thoracic inlet, or parasternal approach. Lesions surrounded by aerated lung may not be visualized due to reflection of the ultrasound beam. Trace pleural effusion or small pulmonary lesions may not be detected, as the entire thoracic cavity is difficult to evaluate with ultrasound (36).

Thoracic CT is a sensitive technique to evaluate for pulmonary metastasis as well as identify abnormalities of the mediastinum, airways, pulmonary parenchyma, and pleural space. When compared to thoracic radiographs, CT eliminates superimposition of structures and has better contrast resolution and detail allowing more specific interpretation of pathology when radiographs are inconclusive or non-specific (33, 37). CT allows for three-dimensional views that provide more information regarding the degree and severity of disease within the pulmonary parenchyma. Unlike radiography, CT can identify foreign body migration tracts as well as provide greater airway detail (32, 37). CT can also identify accessible lesions for CT-guided FNA and biopsy procedures. If a solitary pulmonary mass is found, a CT study can also assess for metastatic disease, evaluate the extent of the mass and also evaluate the thoracic lymph nodes for signs of metastasis, although this can sometimes be difficult due to small size of the lymph nodes (33). If pleural effusion is present and radiographs and ultrasound are non-diagnostic, thoracic CT allows evaluation of the thoracic cavity in greater detail (38). When comparing three-view thoracic radiographs to thoracic CT, CT is more sensitive in detecting pulmonary nodules as it can detect smaller nodules with increased frequency. One study reported pulmonary nodules need to be at least 7–9 mm in diameter to be detected on radiographs while they only need to be 1 mm in diameter to be detected on CT (39). In another study, the smallest nodule detected on radiographs was 3 mm and the smallest nodule detected on CT was 2 mm (34). Metastatic nodules with irregular and poorly defined margins may not be visualized on radiographs and be more reliably seen on CT (39). It can be even harder to detect small to moderate sized pulmonary nodules in large to giant breed dogs on radiographs, making CT a better recommendation for a large breed dogs when ruling out metastatic disease (34). CT must be performed under heavy sedation or anesthesia, so one must ensure the patient is stable to undergo an anesthetic procedure.

## NT-proBNP And Echocardiogram

N-terminal pro-B-type natriuretic peptide (NT-proBNP) measurement can be helpful in differentiating cardiac and respiratory causes of cough in dogs and cats when other diagnostics are ambiguous, especially when acute cough is present (40, 41). In health, atrial monocytes synthesize and store proBNP, which is released into plasma as a result of atrial stretch. ProBNP is also synthesized by ventricular monocytes in patients with chronic cardiac disease and the plasma proBNP concentration reflects the severity of cardiac disease in these individuals. In cats with respiratory clinical signs, plasma NT-proBNP concentrations >270 pmol/L are likely suffering from congestive heart failure (40). In dogs with respiratory abnormalities, a plasma NT-proBNP concentration <800 pmol/L makes respiratory disease more likely the cause of cough and concentrations >1,400 pmol/L raises the probability that the patient is suffering from congestive heart failure (40). NT-proBNP is best interpreted with other diagnostic tests and can be affected by the presence of pulmonary hypertension, systemic hypertension, renal dysfunction, sepsis, and improper handling of blood samples (40). Measurement of NT-proBNP concentration is most helpful when previous diagnostic results are equivocal and the results can provide further information to help support or rule out cardiac disease rather than as an initial diagnostic test. Studies that differentiated respiratory signs caused by primary respiratory tract disease from those caused by congestive heart failure using NT-proBNP did not include a group with cough as the sole clinical sign and most dogs showed at least one and usually several other respiratory abnormalities (stridor, stertor, cough, increased panting, tachypnea, increased respiratory effort, and obvious respiratory distress) (40, 42). Thus, studies that evaluate NT-proBNP specifically related to cough in dogs and cats are lacking. It is also important to note that cats with congestive heart failure do not typically cough, so the diagnostic utility of NT-proBNP may be even more limited in cats with respiratory disease and cough (28).

Along with NT-proBNP, an echocardiogram should be performed when cough is suspected to be caused by cardiac disease. Echocardiography can show whether cardiac changes consistent with congestive heart failure are present but thoracic imaging (radiography or CT) is needed for definitive diagnosis. Echocardiography can detect pulmonary hypertension and masses associated with the heart and surrounding structures as well as provide information about disease processes affecting the pericardial and pleural spaces (28).

## Laryngeal Exam, Endoscopy, And Bronchoalveolar Lavage (Bal)

A relationship between cough and laryngeal dysfunction was found in dogs presenting with cough as the primary problem. Laryngoscopic and bronchoscopic examinations revealed that 19% of dogs with cough also had laryngeal paralysis or paresis without other clinical abnormalities to indicate upper airway disease (43). This study underscores the value of a complete sedated oral exam to evaluate laryngeal function in dogs with cough when no other cause is found on thoracic radiographs.

Endoscopic examination can be performed to visualize respiratory structures and to collect tissue for cytological or histologic examination and samples for culture or biochemical analysis. If a patient is sneezing and has nasal discharge in association with the cough, rhinoscopy using a rigid endoscope is indicated to evaluate the nasal cavity. A flexible endoscope can be retroflexed above the soft palate to evaluate the caudal nasal cavities, choanae, and rostral nasopharynx (30). The flexible endoscope can also be used to evaluate the oropharynx, larynx and large airways. Bronchoscopy allows for evaluation of tracheal collapse, bronchial collapse, bronchomalacia, distal airway collapse, presence of foreign bodies and mucosal lesions, narrowing of airways, and bronchiectasis (29, 43). In dogs with chronic bronchitis, the airway surface is roughened, the glistening character of healthy airway mucosa is absent, the mucosa may be thickened and granular, there is increased airway mucus, the airways appear erythematous, and mucosal vessels may be hyperemic (2, 44). Collapse of the dorsal tracheal membrane is also a common finding and should not be confused with tracheal collapse, which refers to collapse or weakness of cartilaginous tracheal structures. About one third of dogs with chronic bronchitis can have intrathoracic airway collapse during expiration and have a worse prognosis than dogs without intrathoracic airway collapse. In cats with chronic bronchitis, the airways appear narrower than normal. Collapse of the dorsal tracheal membrane and intrathoracic airways are less common in cats than dogs with chronic bronchitis but can occur (2). In dogs with tracheal collapse, typical bronchoscopic findings include gross signs of airway inflammation, excessive airway mucus, and hyperemia. Based on the reduction of tracheal lumen opening during breathing, tracheal collapse can be graded as follows: grade 1 = 25% reduction, grade 2 = 50% reduction, grade 3 = 75% reduction, grade 4 > 90% reduction (31).

In conjunction with a sedated oral exam and/or endoscopy, samples for cytology, histology, and/or culture can be acquired. If the lesion is in an appropriate location, FNA may be performed through the soft palate to obtain samples for cytology or culture. Biopsy instruments via the endoscope can be used to collect samples for histology and culture (30). An endoscopic brush can be used to perform brush cytology by rubbing the brush on the lesion or airway lumen followed by rolling the brush on a glass slide (45). Cytology as well as aerobic, anaerobic, *Mycoplasma* species cultures, and/or polymerase chain reaction should be performed on the BAL fluid to evaluate for inflammatory disease, neoplastic cells, and identify any microorganisms that may be present (29, 32, 46).

## Medical Management of Cough

Options for medical therapy depend on the type of cough and its etiology and include antitussives along with selective use of anti-inflammatories, antihistamines, bronchodilators, and expectorants for specific etiologies (Table 3) (29, 47–53).

**Table 3:** Drugs and dosages to manage respiratory disease (29, 47–53).

	**Drug**	**Dose**	**Route**	**Frequency**
Sedatives	Acepromazine	0.02–0.1 mg/kg (D^*^) 0.56–2.25 mg/kg (D) 0.02–0.1 mg/kg (C^†^) 1–2 mg/kg (C)	IV, IM, SC PO IV, IM, SC PO	q6–8 h q6–8 h q8–12 h q8–12 h
	Butorphanol	0.2–0.4 mg/kg (D) 0.55–1.1 mg/kg (D) 0.2–0.8 mg/kg (C) 1.5 mg/kg (C)	IV, IM, SQ PO IV, SQ PO	q2–4 h q6–12 h q2–6 h q4–8 h
	Midazolam	0.1–0.3 mg/kg (D) 0.05–0.5 mg/kg (C)	IV, IM, SC IV, IM, SC	PRN PRN
	Trazodone	5 mg/kg (D)	PO	q12 h
Antitussives	Butorphanol	0.05–0.1 mg/kg 0.55–1.1 mg/kg	IV, SC PO	PRN q6–12 h
	Hydrocodone^§^	0.22–0.25 mg/kg	PO	q6–8 h
	Codeine	1–2 mg/kg	PO	q6–12 h
	Morphine	0.1 mg/kg	SC, IM	q6–12 h
	Co-phenotrope (diphenoxylate + atropine) Diphenoxylate	0.2–0.5 mg/kg	PO	q12 h
	Dextromethorphan	0.5–1 mg/kg (D) 2–4 mg/kg (C)	PO PO	q8–12 h q8–12 h
Glucocorticoids	Prednisone or prednisolone^**^	0.5–1 mg/kg (D) 1 mg/kg (C)	PO PO	q12–24 h q24 h
	Fluticasone	110–220 mcg	Aerosol	q12 h
Bronchodilators	Theophylline (extended release)	10–20 mg/kg (D) 25 mg/kg (C)	PO PO	q12–24 h q24–48 h
	Aminophylline^††^	10 mg/kg (D) 5–6 mg/kg (C) 2–5 mg/kg	PO PO IV	q6–8 h q12 h q8–12 h
	Terbutaline	0.25 mg/cat 0.625 mg/cat 0.625–5 mg/dog	SC PO PO	q6–8 h q12 h q8–12 h
	Albuterol	0.05 mg/kg (D) 90 mcg	PO Aerosol	q8 h PRN
Antihistamines	Chlorpheniramine	4–8 mg/dog^§§^ 2 mg/cat	PO PO	q8–12 h q12–24 h
	Diphenhydramine	2–4 mg/kg (D)	PO	q8 h
Expectorant	Guaifenesin	3–5 mg/kg	PO	q8 h
Mucokinetic	Acetylcysteine	144 mg/kg (initial dose), followed by 70 mg/kg	IV	q8–12 h
NK_1_ receptor antagonist	Maropitant^***^	1 mg/kg (D) 2 mg/kg (D) 1 mg/kg (C)	SC PO IV, SC, PO	q24 h for up to 5 days or q48 h

* *Dog*.

† Cat.

§ *Caution in cats*.

** *Taper to lowest effective dose*.

†† *Administer over 30–60 min*.

§§ *Maximum dose of 0.5 mg/kg (D)*.

*** *Antiemetic dose. No studies have been performed to establish an antitussive dose*.

## Antitussives

The decision to recommend antitussive treatment is multifactorial and is influenced by the etiology of cough. Factors to consider include: the cause of the cough, the goals of treatment, the impact of cough on the animal's or owner's quality of life, and whether cough is acute or chronic or if the underlying cause can be eliminated. Each of these are important considerations for determining when antitussive treatment is appropriate or when cough suppression would be counterproductive and potentially harmful to the patient. The purpose of antitussive treatment is to reduce the severity and incidence of cough and help prevent compromise of mucociliary defenses (53). Chronic cough can lead to airway damage and irritation, which further stimulates the cough response and leads to a cycle of cough and inflammation that needs to be broken by antitussives (2, 50, 54). Antitussives are specifically indicated when a patient has a non-productive cough or chronic cough that interferes with quality of life. Some dogs become syncopal or are unable to sleep at night from chronic cough, making antitussive therapy essential (2). Dogs with chronic airway disease, including tracheal collapse and chronic bronchitis, or with cough caused by external compression of the trachea or mainstem bronchi are also good candidates for antitussive therapy. When underlying inflammation, infection, or other potential causes of cough have been appropriately addressed and the cough persists, antitussive therapy is appropriate, as cough suppression will not mask underlying disease nor risk harm to the patient. Likewise, treatment is essential for persistent cough that interferes with patient health and lifestyle because it improves comfort and quality of life and may ameliorate progressive airway inflammation (2, 3, 50, 54). In some emergency situations, particularly in cases with airway obstruction due to laryngeal paralysis or tracheal collapse, cough suppression and sedation are indicated if secondary hypoxia, exhaustion, or stress is being induced (29). Antitussives are usually contraindicated in bacterial pneumonia because cough suppression leads to retention of mucus, debris, and other material in the airways preventing clearance of the infection (37).

Opioids reduce cough by decreasing the responsiveness of the cough center to afferent stimuli and also decrease perception of peripheral irritation, exerting their effect via the μ opioid receptors (53, 55). It is suspected that opioids act on sensory nerve endings that initiate cough as well as act within the central nervous system, either at the brainstem respiratory centers or directly on the cough center in the medulla. Mucus production may also be reduced or mucociliary clearance may be increased via μ receptor stimulation, decreasing the need for cough (4). Hydrocodone is the most commonly used opioid in veterinary medicine and is more potent and less sedating than codeine (2, 47, 53, 55). Hydrocodone is usually formulated with homatropine, an anticholinergic drug (47). Co-phenotrope^1^ is a combination of diphenoxylate, an opioid antitussive, and atropine, which decreases mucus secretion and serves as an antimuscarinic bronchodilator. At the muscarinic, cholinergic receptors, atropine displays competitive antagonism of acetylcholine leading to a decrease in vagal transmission causing dilation of both the small and large airways (2). Co-phenotrope is reported to be fairly effective in controlling cough in veterinary patients (50). In general opioids have inconsistent antitussive efficacy and significant adverse effects, especially in cats, including sedation, constipation, respiratory depression, excitement, and muscular spasms (47). It is recommended to start opioid antitussive therapy with a frequent dosing interval and titrate the dose and dosing interval until the lowest effective dose and lowest frequency of administration has been reached (29, 31).

Dextromethorphan is a dextro-isomer of the opiates with receptors in the brain, specifically in the fourth ventricle, and is a common antitussive agent (2). Dextromethorphan can be purchased over the counter and does not typically cause sedation; however, it is much less effective than hydrocodone. The reported antitussive efficacy of dextromethorphan ranges from 50 to 100% of the antitussive efficacy of codeine (2, 53, 55). Anecdotally, in dogs with chronic bronchitis and infectious tracheobronchitis, dextromethorphan has been intermittently effective in reducing cough (2). Dextromethorphan is the safest antitussive for cats (47, 53).

## Glucocorticoids

Glucocorticoids are indicated in dogs and cats with acute or chronic bronchial disease, tracheal collapse, and other respiratory diseases with an inflammatory component (29, 39, 56, 57). In asthmatic cats that are having clinical signs at least twice a week, glucocorticoid therapy is recommended (58). Glucocorticoids inhibit phospholipase A thus inhibiting platelet activating factor, prostaglandins derived from arachadonic acid, and eicosanoids leading to reduced bronchoconstriction, airway edema, and mucus production. Eosinophil chemotaxis and adherence to endothelium is also reduced, further decreasing airway inflammation resulting in a reduction in coughing and improved exercise tolerance (2). The starting dose of glucocorticoid should be sufficient to control inflammation and reduce airway irritation before it is tapered to lowest effective dose. Some patients with chronic airway disease require indefinite corticosteroid therapy but short courses are preferred to limit adverse effects (31, 32). The lowest effective dose needed for cough control should be used since chronic administration or use of a high dose can produce significant adverse effects. Every-other-day dosing over long term use is ideal to minimize adverse effects and maintain a responsive hypothalamic-pituitary axis (32). Prednisone or dexamethasone used at an anti-inflammatory dose are effective in controlling airway inflammation but should be used with caution due to the potential side effects (32, 59). Inhaled corticosteroids such as fluticasone may reduce systemic side effects but cost more than oral glucocorticoids. Fluticasone is the most common inhaled glucocorticoid used to control chronic airway diseases in dogs and cats. A facemask and spacing chamber are needed to administer the inhalant and ensure adequate delivery to the respiratory tract (32). Glucocorticoids should be used with caution in patients that have an active infection, gastrointestinal ulcer, hyperadrenocorticism, diabetes mellitus, hypertension, congestive heart failure, and renal disease. Caution must also be used as glucocorticoids can cause panting and weight gain, both of which impose stress on the respiratory system (29). Because glucocorticoids can mask disease, a definitive diagnosis should be obtained prior to starting the medication (57, 60).

## Antihistamines

Patients with cough that is suspected to be related to rhinitis or sinusitis, post nasal drip, or have an allergic component may benefit from antihistamines (53). Chlorpheniramine is a first generation antihistamine that reduces cough frequency by decreasing cholinergic transmission of nerve impulses. There are multiple probable mechanisms of action including direct and indirect effects peripherally and centrally. Direct effects occur by blocking H1 receptors in the central and peripheral nervous systems. Indirect effects include decreasing mucus secretion and sedative properties. Diphenhydramine has been shown to inhibit citric acid-induced cough in healthy people. First generation antihistamines are also effective in cough management caused by rhinitis via inhibition of histamine and leukotrienes generated by mast cells and eosinophils. Second generation antihistamines do not appear to be effective in reducing cough (61). Sadofsky et al. demonstrated that antihistamines, specifically chlorpheniramine and dexbrompheniramine, inhibit TRPV1 activation thereby demonstrating these drugs may play a role in managing chronic cough (62).

## Bronchodilators

Bronchodilators are indicated when intrathoracic airway collapse, small airway disease, bronchomalacia, and/or expiratory effort are present, but have no effect on the large airways or trachea (29, 31). There are two classes including methylxanthine derivatives (theophylline and aminophylline) and beta-agonists (terbutaline and albuterol) (2). Beta-2 agonists are effective bronchodilators due to their induction of adenylate cyclase activation and secondary increase in intracellular cAMP, which activates protein kinases and leads to a reduction in calcium-dependent coupling of actin and myosin, resulting in relaxation of smooth muscle (2). They also inhibit cholinergic neurotransmission, stabilize mast cell membranes and inhibit release of mast cell mediators, increase mucociliary clearance, and decrease vascular permeability (2). Beta-receptors exposed to a high concentration of agonist undergo phosphorylation and down regulation leading to tachyphylaxis, which is associated with chronic treatment in people but has not been shown to be a clinical problem in dogs and cats (2, 63).

Methylxanthines are phosphodiesterase inhibitors that are well-absorbed in the gastrointestinal tract and cause bronchodilation by decreasing intracellular cAMP degradation leading to bronchial smooth muscle relaxation (2). Diaphragmatic contractility is increased, making it less prone to fatigue in humans, although clinical applicability is uncertain in veterinary patients (2, 64). Methylxanthines also stabilize mast cell membranes, increase mucociliary clearance, and reduce bronchovascular leak (2). A recent study has established that theophylline reduces the excitability of sensory nerves in humans and guinea pigs, thus reducing the cough reflex, which has been previously unrecognized (65). It is not yet known whether these drugs have similar action in dogs or cats. Although bronchodilators are helpful for airway disease management, methylxanthines can cause gastrointestinal upset, cardiac stimulation, and central nervous system stimulation including hyper-excitability in situations where sedation may be more desirable (29, 53).

## Expectorants And Mucokinetics

Coughing patients with large amounts of viscous or inspissated respiratory secretions that cannot be cleared via mucociliary transport may benefit from mucokinetic drugs (51, 53). Mucokinetic drugs facilitate elimination of respiratory secretions, making the secretions more mobile by changing their viscosity, thus having a positive effect on cough (53, 55). Acetylcysteine, a mucolytic drug with anti-oxidant and anti-inflammatory properties, destroys mucoprotein disulfide bonds and produces smaller, less viscous molecules that are unable to efficiently bind inflammatory molecules. Acetylcysteine has anti-oxidant properties, as it is a precursor to glutathione, a free radical scavenger, and also decreases the capability of bacteria to bind to epithelial cells thus reducing the amount of airway bacteria present (53, 66, 67). Acetylcysteine is administered via nebulization but can be irritating to the respiratory mucosa and can induce bronchospasm in cats (51). Acetylcysteine is commonly used in humans and there are anecdotal reports of intravenous use in dogs and oral administration in dogs and cats (51).

While expectorants do not actually suppress cough or alter the viscosity or volume of respiratory secretions, the drugs improve bronchial secretion output, increase clearance of bronchial exudate, and stimulate a more productive cough, which helps remove the mucus from the airways, which eliminates stimuli for cough (51, 67). Mucus secretion stimulation is a reflex action on the gastric mucosa, mediated via the vagus nerve (51). Guaifenesin is an anesthetic muscle relaxant and also acts as an expectorant and mucokinetic agent (51, 68). It is hypothesized that guaifenesin either acts to improve clearance of airway particles or stimulates bronchial secretions through vagal pathways and results in increased volume and decreased viscosity of sputum (68). Overdose of guaifenesin can cause ataxia and sedation (68). Guaifenesin is also a creosote derivative and should be avoided in cats (55, 68).

## Ancillary Therapies

### Oxygen

Dogs and cats that experience a severe acute-onset coughing episode may present cyanotic, and depending on the type of respiratory disease, can also experience syncopal episodes (69). Supplemental oxygen at 40–60% is indicated in hypoxic animals, patients with oxygen saturation <94% on room air or a PaO_2_ <80 mmHg, with an elevated respiratory effort (37). Upon initial presentation, oxygen may be administered via an oxygen mask, hood, cage, or flow-by. If long-term oxygen therapy is indicated, oxygen may be delivered via an oxygen cage, nasal cannula(s), transtracheal method, or intubation for positive pressure ventilation (69).

### Sedatives

Sedation is necessary to decrease the dynamic component associated with upper airway obstruction due to tracheal collapse, brachycephalic airway syndrome, or laryngeal paralysis. Sedatives may also be indicated in patients with severe respiratory distress or patients experiencing paroxysmal coughing. Extreme respiratory difficulty may lead to progressive stress, worsening cough, and respiratory failure thus resulting in rapid progression of clinical signs. Sedation can also be helpful for the owner to administer at home prior to a stressful event that may trigger coughing potentially leading to respiratory distress, especially in patients with tracheal collapse that get worse with excitement. Acepromazine and butorphanol, with or without midazolam or diazepam, are frequently used. Recently, long-term trazodone therapy has become more common (69).

Acepromazine is a phenothiazine that blocks dopamine, exerts alpha blockade effects, and diminishes the ascending reticular activating system. It typically causes mild to moderate, long-acting sedation (70). Acepromazine has marginal respiratory depressant effects. However, acepromazine has long-lasting effects, does not have a specific reversal agent, and suppresses the cardiovascular system; therefore, caution should be used when administering the drug (70, 71). A recent study established that doxapram, a non-selective central nervous system stimulant, at a dose of 1.25 mg/kg is successful in diminishing the sedative effects of acepromazine for at least 30 min (70).

Opioids are recommended drugs for sedation in critical patients as they have little effect on the cardiovascular system and oxygen transport. Butorphanol is a weak μ antagonist and κ agonist opioid, has a short duration of action, and has antitussive properties (47, 71). It also has antiemetic properties and is preferred if the patient is nauseous (71).

Benzodiazepines (e.g., midazolam and diazepam) bind γ-aminobutyric acid and increase the affinity of the receptor. Midazolam tends to be used more often than diazepam in respiratory patients because it can be administered intramuscularly or intravenously, has a more rapid onset and shorter duration of action. In the respiratory patient, benzodiazepines do not provide adequate sedation when used alone and should be given in combination with opioids or acepromazine. It is important to remember these drugs can produce unintended effects of excitement and dysphoria, especially in cats (48).

Trazodone is a serotonin 2A antagonist/reuptake inhibitor and is well-tolerated in dogs for management of canine anxiety disorders (72). Anecdotally, trazodone is useful in dogs with chronic airway disease that tends to flare up with excitement (49).

Although sedation may be vital to alleviate cough and respiratory distress exacerbated by stress and anxiety, close monitoring is imperative as sedation in a patient with an unprotected airway carries a risk for respiratory decompensation and arrest.

### Nebulization

Nebulization and coupage can be beneficial in patients with excessive mucus production and lower airway disease. A nebulizer with a facemask is used to create micro droplets of saline or sterile water, allowing delivery to the lower airways (73). Medications including antibiotics (especially aminoglycosides) and albuterol can be added to the saline, which allows direct delivery of the drug to the lower airways thus reducing systemic side effects (73).

### Management of Environmental Factors

Environmental factors play a large role in some chronic airway disease processes and can cause exacerbation of coughing. Owners should not smoke indoors and any possible airborne irritants such as air fresheners, incense, perfumes, and noxious fumes should be eliminated. Obesity should be controlled, as being overweight increases cough severity and decreases lung function. If an animal has tracheal collapse or other condition associated with tracheal sensitivity, a harness should be used instead of a neck collar (32). Exposure to other animals with potentially infectious diseases should be avoided. Patients should be kept in a cool environment, especially in hot weather. Situations producing excitement, barking, or anxiety should be minimized or avoided entirely (31). Owners should be aware of potential environmental triggers and try to limit exposure as much as possible.

## Other Treatment Considerations

It is not unusual for a patient presented for cough to have a serious, underlying disease process that needs to be immediately addressed. Diuretics and vasodilators are indicated for a patient with congestive heart failure. Pulmonary hypertension secondary to chronic bronchitis requires a phosphodiesterase inhibitor, such as sildenafil or pimobendan. A patient with a pulmonary thromboembolism may require anticoagulant therapy. Cough caused by neoplasia or lung lobe torsion may require surgery for resolution. Antibiotics or antifungals are necessary for cough caused by infectious disease (32). Ultimately, the primary disease process is going to dictate the specific therapy indicated.

## Treatment—Current Research

Chronic cough is a common complaint that interferes with the quality of life of patients as well as owners and treatment is often ineffective. Currently available, effective antitussives are often associated with adverse effects including sedation, constipation, respiratory depression, excitement, muscular spasms, among others. There are antitussives in clinical trials that may be more effective and produce fewer adverse effects than currently available options. For example, the role of transient receptor potential (TRP) channels in cough is an active area of research since many of TRP channel activators are present in respiratory disease and TRP channel expression is greater in airway disease (19, 74). Several of the medications under evaluation or in clinical trials are available for use in veterinary patients.

## Currently Available Therapies For Use In Veterinary Patients

### Neuromodulators

Neuromodulators, including gabapentin, pregabalin, and amitriptyline, have been evaluated in the management of chronic, idiopathic cough. This type of cough may involve a sensory neuropathy and laryngeal irritability. A neuropathy of the recurrent or superior laryngeal nerve has been demonstrated in some patients with chronic, idiopathic cough. Neuromodulating agents, typically used in managing chronic, neuropathic pain, have a benefit in treating patients with chronic cough secondary to neuropathy. An improvement has been demonstrated in quality of life, cough severity, as well as a reduction in cough symptoms with the use of these drugs. Although studies have shown neuromodulating agents aide in treating chronic, idiopathic cough, further studies are needed to demonstrate the optimal dose, time to maximum benefit, symptom relapse rates after treatment, and length of treatment. Further research is also needed in investigating the role of laryngeal neuropathy and which specific patients are most likely to benefit from neuromodulators in cough management (75).

### Maropitant^2^

Maropitant is an NK_1_ receptor antagonist and is a commonly used antiemetic in dogs and cats. SP is a naturally occurring potent NK_1_ agonist, which is inhibited by maropitant (76). Since being approved by the Food and Drug Administration in 2007, the drug has been gaining popularity and anecdotal evidence has shown that it may serve a purpose in treating conditions other than emesis.

SP is distributed throughout the central and peripheral nervous systems. Recent studies have shown immune cells are a chief source of SP, which induces bronchoconstriction, chemotaxis, and neutrophil adhesion to bronchial epithelial-cells, and mast cell degranulation leading to neurogenic inflammatory reactions. In addition, SP plays a role in remodeling the airways during chronic inflammatory disease processes (77). Otsuka et al. compared plasma SP levels in patients with subacute cough and chronic cough to healthy individuals and found elevated plasma SP levels in people with asthmatic and non-asthmatic coughs (78). They also demonstrated that plasma SP levels correlate with airway sensitivity in asthmatic patients establishing SP is involved in multiple origins of cough, although the mechanism of SP may differ depending on the type of cough. There is conflicting evidence in the exact pathophysiology of SP and cough induction; however, evidence shows an association between elevated plasma SP level and airway sensitivity in asthmatic cough (78). Given the inflammatory effects SP has in the respiratory system, maropitant may be a useful antitussive but this remains a topic for future clinical research.

## Novel Therapies Currently Unavailable For Use In Veterinary Patients

### NOP Agonists

Nocioceptin/orphanin FQ peptide (NOP) receptor agonists have shown efficacy in cough treatment. Similar to opioid receptors, NOP receptors are located in the central nervous system as well as the peripheral nervous system. NOP receptors are found on the vagal airway C-fibers in the respiratory system and once activated, inward-rectifying K^+^ channels are triggered, which lead to sensory nerve cell membrane hyperpolarization, thereby reducing tussigenic nerve activity (79, 80). In one study, SCH225288, an NOP receptor agonist, was shown to be a potent and effective antitussive in several animal models, including mechanical-induced cough in cats and infectious tracheobronchitis in dogs (80). Controlled human clinical trials are still needed to evaluate the drug.

### TRPV1 Antagonists

TRPV1 is a non-selective, Ca^2+^-preferring cation channel. This receptor type is found throughout the nervous system, in several organ systems, and is extensively distributed throughout the lungs within vagal C-fiber sensory nerves (81). Studies have shown that TRPV1 is activated by capsaicin, heat, acid, and other mechanisms. Inflammatory mediators can also indirectly activate the TRPV1 channel. TRPV1 is found throughout the lungs, specifically on the sensory airway neurons involved in the cough reflex, and there have been several associations to TRPV1 and cough. In addition, recent studies have proposed that TRPV1 receptors are up regulated in airway inflammation thus decreasing the cough threshold, also known as TRPV1pathy mechanism (82). TRPV1 antagonists may decrease activity of the hypersensitive sensory nerves and lessen the cough reflex response to baseline levels, rather than the increased response seen in diseased states (81).

Tiotropium is a TRPV1 antagonist as well as a muscarinic receptor antagonist and is prescribed for bronchodilation in humans. A recent study has shown that tiotropium acts on the afferent sensory nerves and inhibits TRPV1-mediated effects unrelated to its anticholinergic activity and may also provide some clinical benefit in controlling chronic obstructive pulmonary disease (COPD) and asthma (83). Dexbrompheniramine and chlorpheniramine exhibit antitussive properties by inhibiting TRPV1 activation (62). There are several TRPV1 antagonists undergoing clinical trials that have been shown to reduce the hypersensitivity to capsaicin-induced cough; however, clinical validation has yet to be established (84).

### TRPA1 Antagonists

TRPA1 is expressed on pulmonary C-fibers of the pharynx, larynx, and trachea. A proportion of neurons that express TRPV1 also selectively express TRPA1. TRPA1 is activated by inhaled environmental agents and endogenous tussive molecules, which are produced during inflammatory states (85). Research has shown that cinnamaldehyde, an ingredient from cinnamon and a TRPA1 selective agonist, produces action potentials from bronchopulmonary C-fibers. Oxidative and nitrative stresses also activate TRPA1 channels. The TRPA1 channels have not been analyzed as thoroughly as the TRPV1 channels, but recent studies have revealed a direct role of the TRPA1 pathway in initiating a cough response. HC-03001, a TRPA1 antagonist inhibited a tussive response to cigarette smoke inhalation and also prevented a tussive response to TRPA1 agonists (86). Mukhopadhyay et al. demonstrated *in vitro* confirmation that citric acid directly activates TRPA1 and that GRC 17536, a TRPA1 selective antagonist, has an antitussive effect in a guinea pig cough model. There is a developing link between the vagal TRPA1 receptor and several cough etiologies, suggesting TRPA1 antagonists may serve as potential treatments for chronic cough (85).

### Erdosteine

Erdosteine is a thiol derivative used to treat COPD in humans as well as acute infectious inflammation of chronic bronchitis. The antitussive effects are thought to be related to the drug's anti-inflammatory properties via epithelial cell chemokine reduction and mucociliary clearance enhancement (87).

### Naringin

Naringin is a flavanone derived from citrus fruits and grapes. It has an expectorant effect as well as a peripheral antitussive effect via rapidly acting receptors in healthy guinea pigs. Luo et al. demonstrated naringin also reduces airway hyperresponsiveness in guinea pigs with smoke-induced chronic bronchitis. The drug reduces lung tissue inflammation and tracheal damage in chronic smoke-exposed guinea pigs by decreasing inflammatory activity of infiltrated cells in tissue as well as airway lumen (88).

### Cannabinoid Agonists

Non-selective cannabinoids are currently used to treat a variety of medical illnesses, including nausea, vomiting, pain, glaucoma, anxiety, wasting diseases, and muscle spasms in people. However, significant side effects include mental impairment, psychotropic effects, sedation, and tachycardia, which limit their clinical use. Similar to opioids, research has established that cannabinoids have centrally and peripherally located receptors, CB_1_ and CB_2_, respectively. While CB_1_ receptors are concentrated in the central nervous system, CB_2_ receptors are mainly located on immune tissues including the tonsils, spleen, as well as lymphocytes. Activation of CB_2_ receptors alters the immune system via cytokine release and immune cell migration. Cannabinoid CB_2_ receptor agonist, JWH 133, inhibits sensory nerve function and capsaicin-induced depolarization of the vagus nerve resulting in antitussive activity and inhibition of the guinea pig cough reflex (89). Because CB_2_ receptors are primarily found in the peripheral nervous system, central-mediated side effects seen with non-selective cannabinoids are limited. The development of CB_2_ selective agonists may offer a novel antitussive therapy and treatment for airway inflammation (90).

## Conclusion

Coughing is a common clinical problem in humans and veterinary patients that is difficult to manage and severely impacts quality of life. There are many factors to consider when presented with a coughing patient. Determining if the cough is acute or chronic as well as the etiology of cough is vital in order to determine the best approach for management. Evaluation is done using the combined results from history, physical exam, and a variety of diagnostic tests. Treatment may include management of an underlying disease process with or without cough suppression, immediate treatment of a life-threatening condition, or long term use of antitussive therapy along with other drugs to manage chronic respiratory disease. Recommendations for antitussive therapy in dogs and cats are often subjective having been conveyed in the literature, in some cases for decades, and carried into popular textbooks. Current guidelines are largely based on expert opinion, anecdotal clinical evidence and relatively few rigorous clinical trials. Future research directions include studies into the use of the novel antitussive therapies and specific evaluation of these drugs in dogs and cats. Significant research is needed in veterinary and human medicine to close knowledge gaps in our understanding of cough, including investigations into causes and propagation of the cough response and development of novel and effective antitussive agents.

## Author Contributions

BH conceived the idea, performed the research on the topic, organized, and prepared the manuscript. AB contributed to Table 3, Figure 1, and several manuscript sections. Both authors read, edited, and approved the original manuscript and its revisions.

### Conflict of Interest

The authors declare that the research was conducted in the absence of any commercial or financial relationships that could be construed as a potential conflict of interest.

## Footnotes

^1^Lomotil; Pfizer, New York, NY, USA

^2^Cerenia; Zoetis, Florham Park, NJ, USA
